# Brooklyn, New York foodscape 2007–2011: a five-year analysis of stability in food retail environments

**DOI:** 10.1186/1479-5868-10-46

**Published:** 2013-04-09

**Authors:** Susan Filomena, Kathleen Scanlin, Kimberly B Morland

**Affiliations:** 1 Department of Preventive Medicine, Mount Sinai School of Medicine, One Gustave L. Levy Place, Box #1057, New York, NY, 10029, USA

## Abstract

**Background:**

Food retail studies have focused on the availability of food stores, and on disparities in food access by neighborhood race and income level. Previous research does not address possible changes in local food environments over time, because little is known about the extent to which food environments fluctuate.

**Methods:**

Records of stores licensed to sell food with the New York State Department of Agriculture and Markets from 2007–2011 were compared to detect differences in the total number of food stores and supermarkets annually, as well as the total change for the five-year period. Food stores and supermarkets per 10,000 persons were also calculated. Food retail stability – how many individual food stores opened and closed – was also calculated for total stores and supermarkets. All results were stratified by income level and racial characteristics of 2000 Census Bureau census tracts.

**Results:**

There was an overall increase in all food stores, as well as in supermarkets specifically. However, stability – the proportion of stores that remained open for five years – was greater in higher-wealth and predominantly white areas. Supermarkets remained open in greater proportion than total stores in all racial/ethnic and income areas, but areas with the highest wealth had the greatest supermarket stability. Those areas also had slightly more supermarkets per 10,000 persons, and had no permanent closures of supermarkets. The proportion of new store locations was similar between areas, but lowest-income areas had the greatest proportion of new supermarket locations.

**Conclusions:**

These data suggest that food retail environments change over short periods of time. Stability of food retail environments varies between neighborhoods by race and income. Fluctuations may need to be studied further to understand their impact on food behaviors and health of residents. Finally, the dynamic nature of food retail environments suggests opportunities for policymakers and community organizations to create programs that promote the availability of healthier foods at the neighborhood level.

## Introduction

Researchers have investigated the characteristics of neighborhood-level food environments such as the availability, cost and quality of healthy foods in an effort to understand the complex relationships between these environmental factors and food purchasing, eating, and subsequent health outcomes. A number of studies have documented disparities in the types of food stores located in neighborhoods. Many of these studies investigated differences by income level [1-6] or racial characteristics [4,5] and have generally used cross-sectional study designs. Interpretations of findings may not consider possible instability in food environments – that is, that food retail availability may fluctuate, and may not exert a consistent influence on food behaviors of residents. One study measured the effects of seasonal farmer’s markets on residential access to food retailers over the course of a year [7]; however, we have not identified any study that investigates the consistency of food retailer availability year round.

Understanding fluctuations in local food retail environments can be valuable to further understand how those environments influence the health behaviors of affected residents. If food retail environments are stable, with consistency in volume and types of food stores, this lends support for the assumption that residents are chronically exposed to the features of their food environments. However, if food retail environments are not stable, the changes need to be characterized. For instance, environments where food stores close and are replaced with the same types of food stores may impact residents similarly to stable environments, producing exposures chronically. Alternatively, fluctuations in the volume and types of food stores may create an unpredictable food environment, compelling residents to adopt adaptive behaviors in order to navigate the fluctuations. Additionally, if there are fluctuations in food retail environments, they may open opportunities for food policy to make a positive impact on community health.

We aimed to answer three research questions specifically related to these issues, using five years of data obtained from the New York State Department of Agriculture and Markets (NYSDAM) on the presence of food retailers located in Brooklyn, NY. We aimed to understand: a) if there is variation in the number of all stores selling food, and the number of supermarkets across a five-year period; b) whether any observed variation is associated with neighborhood characteristics; and c) how much food retail environments fluctuate – that is, how often individual retailers opened and closed over the five-year period.

## Methods

### Food store data

The names and addresses of food stores in Brooklyn, New York (Kings County of New York State and a borough of New York City) were obtained annually from NYSDAM from 2007 through 2011. These data represent all stores licensed with the state to sell food in Brooklyn. Each annual file includes the: (a) name of the store; (b) owner’s name; and (c) street address of each business (2007: N = 4,311; 2008: N = 4,333; 2009: N = 4,417; 2010: N = 4,425; 2011: N = 5,431).

Files were cleaned to remove duplicate records and correct for any minor differences between files in the spelling of store names or addresses that indicated the records represented the same store. Secondly, the records were reviewed to identify and code supermarkets. For the purpose of this study, supermarkets were defined as large chain food stores and food stores that require membership (i.e. food co-operatives and Costco). They were identified by name recognition and coded electronically. Stores not identified as supermarkets comprised a heterogeneous group of retailers selling food, including small corner stores/bodegas, convenience stores, fruit and vegetable markets, dollar stores, pharmacies, and specialty stores.

Because name recognition could not be used to identify other types of food stores with accuracy, only supermarkets were coded. Analyzing supermarkets apart from other stores is beneficial because supermarkets are the primary food outlet for shoppers more often than smaller stores, and supermarkets are associated with increased availability of healthier foods [4,8], and with lower prices for healthier foods [9].

#### Definitions of store changes

Five-year differences were defined as the differences in the total number of food stores in the 2011 NYSDAM database compared to the 2007 database. To determine differences across the five years, three variables in the NYSDAM databases were used: 1) store name; 2) address; and 3) owner name.

*Stability* of individual stores was determined by comparing addresses and store names in the 2007 and 2011 databases. Stores were determined to be the same store if they had the same name and address, even if there were spelling or punctuation differences. If there was a minor difference in a store name listed at the same address in the two databases, (i.e. the addition of a word such as “Gourmet”), then the owner names for each year were compared to determine whether the store had transferred ownership. If the owner names were different, they were considered different stores; if they were listed under the same owner, they were considered to be the same store.

Once the two databases were matched by store name and address, two categories were created to define 2007 stores as open or closed in 2011. All 2007 stores were coded into one of these categories. *Closed stores* were given a sub-code to indicate whether they were: (a) replaced by a different store that also retailed food; or (b) vacant or occupied by a non-food retailer (in which case their addresses did not appear in the 2011 NYSDAM database). A final category was created for *stores at new addresses* appearing in the 2011 NYSDAM file that were not in the 2007 NYSDAM file. The definitions for each of these categories are described below.

•***2007 stores remaining open through 2011 (“Stable Stores”):*** Stores that matched on both store name and address in the 2007 and 2011 databases.

•***2007 stores closed by 2011 (“Closed Stores”):*** Stores in the 2007 NYSDAM database that were not matched to store name and address in the 2011 NYSDAM database.

∘ ***Food store replacements (“Replaced Stores”):*** Stores in the 2007 database that did not match on store name but the address remained in the 2011 database with a different food store name.

∘ ***Not replaced by a food store as of 2011 (“Permanent Closures”):*** Stores in the 2007 database that did not match to name or address in the 2011 NYSDAM database. Since the NYSDAM databases contain all businesses licensed with the State of New York to retail food, it is assumed that if a store name and address listed in the 2007 database is not in the 2011 database, then the store was either vacant or was occupied by a business that does not retail food in 2011.

•***2011 food stores in new locations (“New Locations”):*** Stores in the 2011 NYSDAM file that were not matched to store name and address in the 2007 NYSDAM file.

#### Geocoding

Once files were cleaned and coded, addresses were geocoded using ArcGIS v9.3 (Environmental Systems Research Institute (ESRI); Redlands, CA). Store addresses were then joined to shape files of 2000 United States Census Bureau census tracts for Brooklyn, NY, obtained from the New York City Department of City Planning (DCP) (http://www.nyc.gov/html/dcp/html/bytes/applbyte.shtml).

### Census data

The 2000 United States Census Bureau census tracts were categorized by neighborhood wealth and racial/ethnic characteristics, using census data for annual household median income, white non-Hispanic population, and total population. Census tracts were categorized by area wealth into the following groups: *lowest income:* < $25,000; *middle income*: $25,000-49,999; and *highest income:* > = $50,000 (top range $93,327). Area racial/ethnic characteristics of census tracts was determined by the proportion of white population: *predominantly non-white:* < 25% white; *racially mixed:* 26-75% white; and *predominantly white:* >75% white.

### Exclusion criteria

Census tracts were excluded if there was no population (N = 8), because race and income analyses could not be performed. Additionally, one census tract was excluded because its median household income was an outlier at $116,599. Store records were excluded if they were determined to be duplicate records or non-exact address matches in ArcGIS, resulting in the final store datasets for each year (2007: N = 4,038; 2008: N = 4,048; 2009: N = 4,164; 2010: N = 4,284; 2011: N = 5,145).

### Statistical analysis

The total population for Brooklyn was obtained from the 2000 Bureau of the Census. Total population of each tract was summed to determine the total population for each racial/ethnic and income subgroup. Food stores and supermarkets per 10,000 persons were calculated for each subgroup in each year by dividing the number of stores by the subgroup population, then multiplying by 10,000. The number of *stable stores* was calculated by summing the number of stores in the 2007 NYSDAM database that matched stores in the 2011 database; the number of *closed stores* was calculated by subtracting the number of stable stores from the number of total stores in the 2007 NYSDAM database; the number of *permanent store closures* was calculated by summing the addresses in the 2007 database that were not in the 2011 database. The number of *closed stores that were replaced by other food stores* was calculated by subtracting the number of permanent closures from the total number of closed stores; and the number of *new food store locations* was calculated by first subtracting the number of stable store from the number of 2011 total stores, (to get the total number of new stores, which includes replaced stores and new locations), then subtracting the number of replaced stores from that. Percentage calculations were made to describe proportions of stable stores, permanent closures, and new store locations in the text, but percentages are not displayed in the tables. All calculations were made in SAS v 9.3 (SAS Systems, Cary NC).

## Results

### Brooklyn population and density of stores

A total of 774 Brooklyn census tracts, with a total of 66.94 square miles, were included in analysis. The total population for Brooklyn, New York is almost 2.5 million, with an average of 36,829 persons per square mile. For the purpose of clarity throughout the results section, food store density will be discussed as stores per 10,000 persons. Overall in Brooklyn, there were about 16 food stores and 0.7 supermarkets per 10,000 residents in 2007 (Table 1). There was an annual increase in the number of total stores through 2011, with the greatest increase taking place between 2010 and 2011. The total increase, or 5-year change, was 1107 total stores (an additional 4.5 stores per 10,000 persons) and 16 supermarkets (0.1 supermarkets per 10,000 persons).

**Table 1:** Number (n) of people, census tracts and all food stores in Brooklyn, New York by area income and race/ethnicity, 2007-2011

		**Income**			Race*		
	**Brooklyn**	**Lowest**	**Middle**	**Highest**	**Predominantly non-white**	**Racially mixed**	**Predominantly white**
**Total population N (%)**	2,465,326 (100.0)	653,652 (26.5)	1,544,649 (62.7)	267,025 (10.8)	1,062,743 (43.1)	831,362 (33.7)	571,221 (23.2)
**Census tracts N (%)**	774 (100.0)	181 (23.4)	477 (61.4)	116 (15.0)	311 (40.2)	264 (34.1)	199 (25.7)
Total food stores** N (Number of stores per 10,000 persons)							
**2007**	4038 (16.4)	1128 (17.3)	2614 (16.9)	296 (11.1)	1624 (15.3)	1557 (18.7)	857 (15.0)
**2008**	4048 (16.4)	1140 (17.4)	2612 (16.9)	296 (11.1)	1631 (15.4)	1545 (18.6)	872 (15.3)
**2009**	4164 (16.9)	1170 (17.9)	2695 (17.5)	299 (11.2)	1682 (15.6)	1596 (19.2)	1596 (19.2)
**2010**	4284 (17.4)	1189 (18.2)	2791 (18.1)	304 (11.4)	1775 (16.7)	1621 (19.5)	908 (15.9)
**2011**	5145 (20.9)	1459 (22.3)	3297 (21.3)	389 (14.6)	1983 (18.7)	2134 (25.7)	1028 (18.0)
**5- Year change**	1107 (4.5)	331 (5.1)	683 (4.4)	93 (3.5)	359 (3.4)	577 (6.9)	171 (3.0)
**Supermarkets N (Number of stores per 10,000 persons)**							
**2007**	173 (0.7)	43 (0.7)	107 (0.7)	23 (0.9)	88 (0.8)	59 (0.7)	26 (0.5)
**2008**	171 (0.7)	45 (0.7)	102 (0.7)	24 (0.9)	84 (0.8)	62 (0.7)	25 (0.4)
**2009**	189 (0.7)	45 (0.7)	105 (0.7)	23 (0.7)	89 (0.8)	59 (0.7)	25 (0.4)
**2010**	187 (0.8)	50 (0.2)	112 (0.7)	25 (0.9)	96 (0.9)	62 (0.7)	29 (0.5)
**2011**	189 (0.8)	53 (0.8)	109 (0.7)	27 (1.0)	98 (0.9)	62 (0.5)	29 (0.5)
**5- Year change**	16 (0.1)	10 (0.2)	2 (0.01)	4 (0.1)	10 (0.1)	3 (0.4)	3 (0.1)

*N* number; % percentage.

*Area Race: Predominantly Non-White: <25% White non-Hispanic; Racially Mixed: 25-75% White non-Hispanic; Predominantly White: >75% White non-Hispanic.

** Total Food Stores are any of the following: supermarkets (chain, and stores requiring membership), small grocers, bodegas/corner stores, convenience stores, specialty shops, fruit and vegetable markets, dollar stores and pharmacies.

There is some variation in the availability of food stores by area wealth. The density of total food stores was highest in the lowest-income areas, across all years, although middle-income areas had the greatest total gain (683, compared with 331 in the lowest-income areas and 93 in the highest-income areas) over five years. However, the density of supermarkets was slightly greater each year in the highest-income versus other income areas (Table 1). Also, supermarkets comprised a greater proportion of all stores in the highest-income areas compared with the other income groups (7%, versus 3% in middle-income and 4% in lowest-income areas).

Regarding food store availability by racial characteristics of census tracts, there was a higher density of total food stores in racially mixed areas compared with predominantly non-white areas and predominantly white areas, consistent across all years. The greatest increase in total stores was observed in racially mixed areas. The increase in supermarkets was similar across areas (Table 1). A higher proportion of stores in predominantly non-white areas were supermarkets (5%) compared with both mixed (3%) and predominantly white (3%) areas.

### Food store stability by area wealth

Table 2 shows the types of food store change used to characterize food retail stability, for all of Brooklyn and categorized by income of census tracts. Of the 4,038, see Table 1, food stores available in Brooklyn in 2007, 2,149 were still open in 2011 (“Stable Stores”). Almost half of 2007 stores (1,889) (see Table 1) were closed by 2011 (“Closed Stores”). Of these, 1,193 were replaced by a new food store at the same address (“Replaced Stores”), but 696 were not replaced by a food store – their addresses are either vacant, or occupied by other types of stores (“Permanent Closures”). Of the 5,145 stores in 2011, 1,803 (35%) were opened in locations that did not have a food store in 2007 (“New Locations”).

**Table 2:** Stability of food stores over 5 years, with number and density per 10,000 persons, by median income of census tract

	**Total**		**Lowest income**		**Middle income**		**Highest income**	
	**Total stores**	**Supermarkets**	**Total stores**	**Supermarkets**	**Total stores**	**Supermarkets**	**Total stores**	**Supermarkets**
**2007 stores remaining open through 2011 (“Stable Stores”)**	2149 (8.7)	131 (0.5)	561 (8.6)	31 (0.5)	1393 (9.0)	79 (0.5)	195 (7.3)	21 (0.8)
**2007 stores closed by 2011 (“Closed Stores”)**	1889 (7.7)	42 (0.2)	567 (8.7)	12 (0.2)	1221 (7.9)	28 (0.2)	101 (3.8)	2 (0.07)
**Food store replacements (“Replaced Stores”)**	1193 (4.8)	35 (0.1)	370 (5.7)	10 (0.2)	765 (5.0)	23 (0.2)	58 (2.2)	2 (0.07)
**Not replaced by a food store as of 2011 (“Permanent Closures”)**	696 (2.8)	7 (.03)	197 (3.0)	2 (0.03)	456 (3.0)	5 (0.03)	43 (1.6)	0
**2011 food stores in new locations (“New Locations”)**	1803 (7.3)	23 (0.1)	528 (8.1)	12 (0.2)	1139 (7.4)	7 (0.2)	136 (5.1)	4 (0.1)

*N* number.

Total Stores includes: supermarkets (chain, and stores requiring membership), small grocers, bodegas/corner stores, convenience stores, specialty shops, fruit and vegetable markets, dollar stores and pharmacies.

A higher degree of stability is observed for supermarkets throughout Brooklyn: 131 of the 173 supermarkets in 2007, Table 1, remained open through 2011 (76%). Forty-two of the 2007 supermarkets were closed by 2011 and 35 were replaced by another supermarket. Seven closed supermarkets were not replaced by a supermarket or another type of food store; however, there were 23 new locations of supermarkets in Brooklyn by 2011 (Table 2).

In terms of stability of individual stores by area wealth, the lowest**-**income areas had the least stability – of the 1,128 2007 stores in these areas (Table 1), only 561 remained open through 2011 (Table 2). This represents 50% of total 2007 stores, compared with 53% in middle-income and 66% in the highest-income areas. Similarly to Brooklyn in general, individual supermarkets had greater stability than total stores across all income areas. However, the highest-income areas had the greatest supermarket stability (91% stable supermarkets compared with 72% in lowest-income areas and 74% in middle-income areas). Further, highest-income areas had no permanent closures of supermarkets, while there were small numbers of these in the lowest-income (N = 2) and middle-income (N = 5) areas (Table 2). The proportion of 2011 stores in new locations was comparable between wealth areas, but new supermarket locations were greatest in lowest-income areas (23% compared with 6% in middle-income and 15% in highest-income areas).

### Food store stability by area racial characteristics

Table 3 shows food retail stability by area racial/ethnic characteristics. Of the 857 stores open in 2007 in predominantly white areas (Table 1), 538 were still open in 2011 (Table 3) – 63% stable stores compared with 54% in mixed areas and 48% in predominantly non-white areas. The same trend is seen for supermarkets, where 23 out of 26 stores were still open in 2011 (Table 3) – 88% stable supermarkets compared with 76% in mixed population areas, and 72% in predominantly non-white areas. The proportion of total new store locations was greatest in mixed population areas, but the proportion of new supermarket locations was slightly higher in predominantly white areas (14%, compared with 13% in predominantly non-white areas and 10% in racially mixed areas). The number of permanent closures was highest in mixed population areas: 297 stores closed permanently, compared with 258 in predominantly non-white areas and 141 in predominantly white areas (Table 3). There were also small but similar numbers of permanent supermarket closures in each area by racial characteristics.

**Table 3:** Stability of food stores over 5 years, with number and density per 10,000 persons, by race/ethnicity of census tract

	**Predominantly non-white**		**Racially mixed**		**Predominantly white**	
	**Total stores**	**Supermarkets**	**Total stores**	**Supermarkets**	**Total stores**	**Supermarkets**
**2007 stores remaining open through 2011 (“Stable Stores”)**	778 (7.3)	63 (0.6)	833 (10.0)	45 (0.5)	538 (9.4)	23 (0.4)
**2007 stores closed by 2011 (“Closed Stores”)**	845 (8.0)	25 (0.2)	724 (8.7)	14 (0.2)	319 (5.6)	3 (0.1)
**Food store replacements (“Replaced Stores”)**	587 (5.5)	22 (0.2)	427 (5.1)	11 (0.1)	178 (3.1)	2 (0.04)
**Not replaced by a food store as of 2011 (“Permanent Closures”)**	258 (2.4)	3 (0.03)	297 (3.6)	3 (0.04)	141 (2.5)	1 (0.02)
**2011 food stores in new locations (”New Locations”)**	618 (5.8)	13 (0.1)	874 (10.5)	6 (0.1)	312 (5.5)	4 (0.1)

*N* number.

Area Race: Predominantly non-White: <25% White non-Hispanic; Racially Mixed: 25-75% White non-Hispanic; Predominantly White: >75% White non-Hispanic.

Total Stores are any of the following: supermarkets (chain, and stores requiring membership), small grocers, bodegas/corner stores, convenience stores, specialty shops, fruit and vegetable markets, dollar stores and pharmacies.

## Discussion

Brooklyn is a highly populated urban setting with a high density of food stores, of which supermarkets comprise a small proportion – 4% on average. The food retail environment in Brooklyn is dynamic: there was an overall increase in total stores and supermarkets throughout Brooklyn, but there were also many store closures during the five-year period. These closures were offset by food store replacements as well as store openings in new locations (including 23 new supermarkets). Stability of the food retail environment varied by wealth and racial/ethnic make-up of census tracts. Differences were observed in the density of stores, proportion of supermarkets, and individual store stability (proportion of stores that were open for the entire five-year period). Although there were fewer stores per 10,000 people in the highest-income and predominantly white areas, store stability was higher in these areas. Individual supermarkets were more stable than the general food retail environment in all race and income areas, yet supermarket stability followed a similar pattern – fewer supermarkets closed in higher-income areas. While the proportion of all 2011 stores in new locations was similar throughout race areas and income areas, new supermarket locations in the lowest-income areas were disproportionately high compared with other income groups. However, proportion of all stores that were supermarkets in 2011 was still higher in the highest-wealth areas.

The reasons for the observed differences between neighborhoods and the fluctuations in the food retail environment cannot be determined using the data provided. These data represent a specific period, which encompassed an economic recession in the United States [10]. These economic factors may have influenced the stability of the food retail environment. For example, it is possible that a decrease in real estate value influenced the number of store openings. However, since lower-income neighborhoods experienced greater instability in the food marketplace (more store closings and openings), one could surmise that businesses in those neighborhoods had fewer resources to help them withstand economic downturns than those in the highest wealth areas, and many could not survive for long. Although the trends observed in Brooklyn during the study period may not be generalizable to any other five-year period, the findings are important for considering the impact of food retail fluctuations on the shopping patterns of affected residents.

The most salient data presented here is the amount of fluctuation in neighborhood food retail, and the varying degrees of stability between areas by wealth and racial characteristics. The findings highlight the need to understand how consumers utilize adaptive behaviors, or modify their food shopping patterns, to navigate a frequently changing environment. In neighborhoods where stores often change ownership or close permanently, residents may change their shopping behaviors in any number of ways. They may spend more time adjusting their shopping routines than those in more stable food environments. The adjustments necessary are not only geographic (finding the next best location to shop), but also economic (adjusting to different prices at the new shopping location), and dietary (adjusting to varying selections at other stores). Where closed food stores are replaced, access is restricted in the interim between a closing and an opening. Permanent closures of supermarkets in particular disrupt residents’ access to food [11-13]. While permanent supermarket closures were much lower than that of total stores, the closing of just one supermarket – where the next nearest is several blocks away or in a different neighborhood – might mean having to find transportation and additional time to travel farther to shop. Further, older and less ambulatory residents affected by a supermarket closing may begin relying on smaller stores – which likely offer a comparably limited range of goods at higher prices. In this study, all area types except highest-income areas were affected by permanent closures of supermarkets.

Researchers, policymakers, and others might consider how they can utilize fluctuations in the food retail environment to further the goal of improving access to healthy foods. For example, these data demonstrate that non-supermarket food stores are the most prevalent and have the highest turnover. The turnover is particularly pronounced in the lowest-income study areas and in predominantly non-white and mixed population areas. On one hand, these fluctuations may create unpredictable shopping environments for neighborhood residents. On the other, fluctuation – combined with the high prevalence of non-supermarket stores – may indicate tremendous opportunity for public health policy to work with store owners and community partners to influence the availability of healthy foods in these neighborhoods, as has been suggested by others [14-17]. When a food store changes hands or simply upgrades its image, the owner may be open to new ideas. Also, such changes may be promising to consumers who are looking to broaden their options. Therefore, fluctuations in the food retail environment may provide an open window to reach both store owners and residents.

This analysis has some limitations, which should be considered when interpreting the findings. First, for race and income analyses we used Census 2000 data and therefore were not able to consider demographic changes that occurred in these neighborhoods during the study period. The reason for this was that by the 2010 census, some census tract boundaries had changed. Second, we used government data to identify food stores. While these data are updated regularly, they do not include store type categories, which limited our analysis to total stores and stores validly identified as supermarkets. Third, this analysis is limited to the *number* of stores where food is available, without investigating the availability of certain food items at those stores, or their prices and quality. Several studies have investigated these factors and found that 1) smaller stores and low-income neighborhoods are associated with poorer quality of produce [18,19] and 2) food stores located in low-income and neighborhoods of color have lower availability of food items considered healthier, as well as higher prices for these items [5,9,20]. Other work has described the impact of food quality on purchasing behavior [21-23]. Finally, since an urban foodscape differs from other environments in terms of the density of population and stores, our findings may not be generalizable to other areas of the United States or internationally.

Despite these limitations, the data presented are to our knowledge the first to demonstrate the dynamic nature of food retail environments, the data represent, the entirety of one urban setting and not a sample, enabling a comparison of neighborhoods by race and income that illuminates disparities for some residents. As in other urban settings, the dense population of Brooklyn (nearly 2.5 million people) underscores the need to understand how residents are affected by fluctuations in their food environments. In Brooklyn, there are over one million residents in predominantly non-white neighborhoods, and 650,000 residents in the lowest-income neighborhoods – areas that these data show are the least stable food retail environments.

## Conclusion

This analysis demonstrates that neighborhood food retail environments experience fluctuations, and therefore may not exert a consistent influence on the behaviors and health of residents. The documented variations in foodscapes reveal the need for researchers to develop methods for characterizing the stability of food retail environments in order to better understand the influence of food store fluctuations on purchasing patterns, as well as to interpret associations between food environment and health outcomes. In these data, areas with the most stable food retail environments are the least densely populated. Meanwhile, the most unstable food retail environments affect over one million people in non-white areas and 650,00 in the lowest-income areas. Finally, the degree of fluctuation seen in the Brooklyn food retail environment points to an opportunity for public health policymakers and city planners to work with store owners and communities to develop more sustainable programs that market fresh, affordable foods that promote health.

## Competing interests

The authors declare that they have no competing interests.

## Authors’ contributions

SF is the primary author and led the analysis and writing. KS contributed to interpretation of the data and writing. KM conceived the manuscript and contributed to analysis, interpretation of the data and writing. All authors have read and approved the final manuscript.

## Authors’ information

SF and KS are research coordinators at the Mount Sinai School of Medicine. KM is an environmental epidemiologist and associate professor at the Mount Sinai School of Medicine.
